# European Bat *Lyssavirus* Infection in Spanish Bat Populations

**DOI:** 10.3201/eid0804.010263

**Published:** 2002-04

**Authors:** Jordi Serra-Cobo, Blanca Amengual, Carlos Abellán, Hervé Bourhy

**Affiliations:** * Universitat de Barcelona, Barcelona, Spain; † Institut Pasteur, Paris, France; ‡ Ministerio de Sanidad y Consumo, Madrid, Spain

**Keywords:** Lyssavirus, polymerase chain reaction, serology, Spain

## Abstract

From 1992 to 2000, 976 sera, 27 blood pellets, and 91 brains were obtained from 14 bat species in 37 localities in Spain. Specific anti-*European bat lyssavirus 1* (EBL1)-neutralizing antibodies have been detected in *Myotis myotis, Miniopterus schreibersii, Tadarida teniotis,* and *Rhinolophus ferrumequinum* in the region of Aragon and the Balearic Islands*.* Positive results were also obtained by nested reverse transcription-polymerase chain reaction on brain, blood pellet, lung, heart, tongue, and esophagus-larynx-pharynx of *M. myotis*, *Myotis nattereri, R. ferrumequinum,* and *M. schreibersii*. Determination of nucleotide sequence confirmed the presence of EBL1 RNA in the different tissues. In one colony, the prevalence of seropositive bats over time corresponded to an asymmetrical curve, with a sudden initial increase peaking at 60% of the bats, followed by a gradual decline. Banded seropositive bats were recovered during several years, indicating that EBL1 infection in these bats was nonlethal. At least one of this species (*M. schreibersii*) is migratory and thus could be partially responsible for the dissemination of EBL1 on both shores of the Mediterranean Sea.

Rabies is a worldwide zoonosis due to *Lyssavirus* infection; multiple host species act as reservoirs. This disease infects the central nervous system of humans and other mammals. Bats are no exception, as proved by the 630 positive cases detected in Europe from 1977 to 2000 (*1*,*2*). Recent molecular studies have shown genetic differentiation in lyssaviruses that cause rabies among European bats, leading to a classification into two new genotypes, 5 and 6, which correspond to *European bat lyssavirus 1* (EBL1) and EBL2, respectively (*3*,*4*). As a result of a recent molecular study, two new lineages within genotype 5 have been identified—EBL1a and EBL1b; the latter is potentially of African origin, which suggests south-to-north transmission (*5*). However, despite molecular advances and many European cases verified to date, knowledge of the prevalence and epidemiology of EBL is limited. Of the 30 insectivorous bat species present in Europe, approximately 95% of cases occur in the species *Eptesicus serotinus* (*2*). This species, which is nonmigratory, cannot be linked to all the different foci of positive cases in Europe (*6*). In Spain, the first case of bat lyssaviruses was recorded in 1987 in Valencia. Sixteen more cases were reported in *E. serotinus* (*7*). The distribution of positive cases in Spain is indicated in Figure 1.

**Figure 1:**
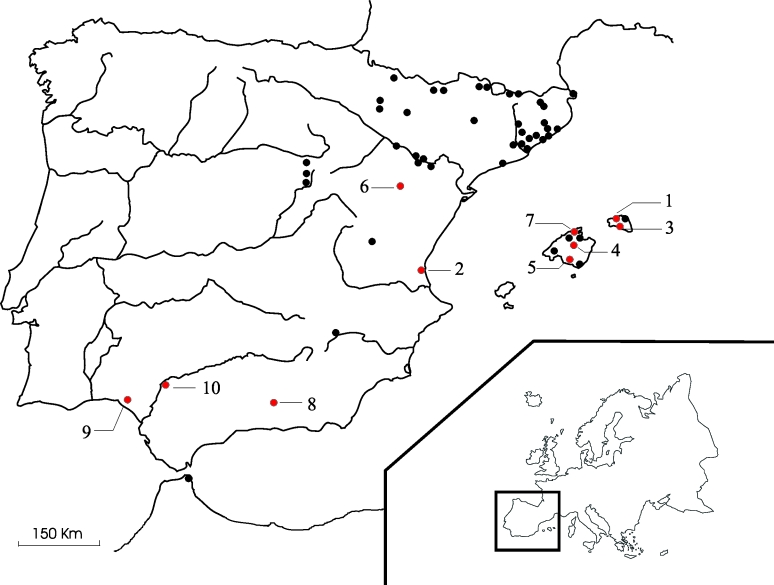
Map showing the localities in Spain where bats have been analyzed***.*** 1. Ciutadella; 2. El Saler; 3. Ferreries; 4. Inca; 5. Llucmajor; 6. Oliete; 7. Pollença; 8. Granada; 9. Huelva; 10. Sevilla. Points in red indicate colonies where positive results were obtained according to our study (Localities Nos. 1, 3, 4, 5, 6, and 7) and previous studies (Localities Nos. 2, 8, 9, and 10) (*7*).

Recently, clinically silent rabies infection has been reported in zoo bats (*Rousettus aegyptiacus)* in Denmark and the Netherlands (*8*). This observation, together with the results of an experimental challenge, suggests that this frugivorous bat species of African origin can survive EBL1 infection or inoculation (*9*). Silent infection has also been described in the American bat (*Tadarida brasiliensis mexicana)* (*10*,*11*) and suggests an alternative viral strategy for *Lyssavirus* infection of European insectivorous bats compared with the terminal infection commonly associated with rabies infection.

To investigate these observations, a 9-year study was undertaken in Spain to locate and determine the colonies and species of bats carrying EBL or *Lyssavirus* antibodies, monitor the prevalence of seropositive bats, and characterize circulating lyssaviruses.

## Material and Methods

### Selection of Bat Colonies and Banding

The study area consisted mainly of the Spanish Autonomous Regions of Aragon, Balearic Islands, Catalonia, and Valencia (Figure 1) (*12*–*15*). The region of Ceuta (North Africa, near the Straits of Gibraltar) was also studied because of its proximity to Europe. Bat colonies were selected according to the following criteria: colony behavior (anthropophilic, migratory, gregarious) and proximity of the colonies to urban areas. The Valencia bat colony was widely sampled because the first case of bat *Lyssavirus* in Spain was reported there (*7*). Colonies exhibiting positive sera were more intensively explored during the years after the first detection. From 1996 to 2000, bats from the locations Nos. 4, 5, and 7 were banded in the forearm to facilitate monitoring of their movements between colonies (*16*).

### Blood Sampling

To draw blood, we set the bat face upward with a stretched wing. The patagium was wiped clean and locally disinfected with a sanitary towel soaked in 96% alcohol to prevent infections. Immediately afterwards, a small puncture was made next to the radius proximal epiphysis. Blood was collected in an Eppendorf vial by using a Pasteur pipette. The amount of blood sampled varied from 0.2 mL to 0.5 mL, according to the size of the animal. A sterilized absorbent hemostatic sponge impregnated with gelatin was administered to prevent bleeding and facilitate healing. Pressure was applied to the wound with a sanitary towel for 30 seconds. The bats were given 10% glucose water to drink to prevent dehydration and provide rapidly assimilated compounds for energy. Once bleeding ceased, the bat was released. Vials containing blood were stored at 4ºC for a few hours. Samples were centrifuged for 20 minutes at 5,000 rpm, and the serum was extracted with a pipette. Serum samples and blood pellets were stored at –20°C.

### Detection of EBL Antibodies

The technique used for the detection of EBL antibodies is an adaptation of the Rapid Fluorescent Focus Inhibition Test (*17*). A constant dose of a previously titrated, cell culture-adapted EBL1 challenge virus 8918FRA (*5*) was incubated with threefold dilutions of the sera to be titrated. After incubation of the serum/virus mixtures, a suspension of BSR (a clone of BHK-21) cells was added. After 24 hours’ incubation, the cell monolayer was acetone-fixed and stained with a fluorescent anti-nucleocapsid antibody (Bio-Rad, Marnes-la-Coquette, France) to detect the presence of non-neutralized virus (fluorescent foci). Titers are presented as an arithmetic mean of two independent repetitions. Serum samples with antibody titers <27 are considered negative for EBL1-neutralizing antibodies. The percentages of seropositive bats and the years in which bats were analyzed (from 1996 to 2000) were correlated, and regression curves were obtained. To confirm the specificity of the reaction, the same test was performed on selected sera by using the challenge virus strain (CVS) (*17*) and 9007FIN EBL2 challenge viruses (*5*).

### Brain Sampling

Brain samples were obtained from dead bats, submitted by citizens. Dead bats found in the studied refuges were also gathered. The bats found dead from 1994 to 1996 were analyzed by direct immunofluorescence technique (*17*,*18*). The bats found dead from 1997 to 2000 were analyzed by nested reverse transcription-polymerase chain reaction (RT-PCR) (*9*). To eliminate cross-contamination at necropsy, sterilized instruments were used.

### Detection of EBL Antigens

The standard fluorescent antibody test (FAT) was performed on brain tissue specimens of the bats by using the polyclonal fluorescein isothiocyanate-labeled rabbit anti-rabies nucleocapsid immunoglobulin G, as described by the manufacturer (Bio-Rad). Brain smears obtained from noninfected and CVS-infected mice were incorporated as controls in each FAT test run.

### Detection of EBL1 RNA

Total RNA was extracted from tissue samples (50 mg -100 mg) by using the TRIzol method (Invitrogen, Groningen, the Netherlands), purified with chloroform and precipitated with iso-propanol (Merck, Darmstadt, Germany). After being washed with 70% ethanol, the RNA pellet was dried, resuspended in a volume of 50 μL bidistilled water and stored at –20ºC. cDNA synthesis of the genomic and antigenomic sense of the EBL1a nucleoprotein RNA was performed by annealing, at 70ºC for 3 minutes, 2 μL of total RNA extract with 15 pmol of primers N60 (5’-TCCATAATCAGCTGGTCTCG-3’, positions 98-117, relative to rabies genome) (*19*) and N41, as described previously (*5*).

Amplification of 5 μL of the cDNA template was performed in a final volume of 50 μL containing 1x magnesium-free PCR buffer (Invitrogen), 5 mM deoxynucleoside triphosphate (NTP) mix (containing 1.25 mM each of dATP, dCTP, dGTP, and dTTP), 5 mM magnesium chloride (Invitrogen), 2 U *Taq* DNA polymerase (Invitrogen), and 30 pmol of primers N60 and N41. The amplification was performed on a GeneAmp PCR System 9700 Thermal cycler. The program started with one denaturation step at 94ºC for 5 minutes, followed by 30 cycles of 94ºC for 30 sec, 60ºC for 30 sec, and 72ºC for 40 sec. The amplification was finalized by an ultimate elongation step at 72ºC for 5 min. The primary amplification products were stored at –20ºC. For nested RT-PCR, the amplified product was diluted 10 times in distilled water. Then the second amplification was performed as described above with the following modifications: 30 pmol of primers N62 and N63 (N62: 5’-AAACCAAGCATCACTCTCGG-3’, position 181-200; N63: 5’-ACTAGTCCAATCTTCCGGGC-3’, position 342-323 relative to the *Rabies virus* genome) (19) were used, and the elongation steps were performed at 72ºC for 30 sec. Aliquots (5 µL) of nRT-PCR products were analyzed by horizontal agarose (1.5%) gel electrophoresis. Gels were stained with 1 µg/mL ethidium bromide and photographed under UV light.

Extraction of RNA was performed in a level-2 biosafety laboratory. Then we prepared the template and RT-PCR mix and added DNA to the mix with aerosol-resistant tips in two different rooms. We also performed nRT-PCR on tissue RNA, omitting reverse transcriptase. Positive (isolate no. 2002FRA) and negative (H_2_O) controls were incorporated into each of the following steps: total RNA extraction, cDNA synthesis, and each of the two steps of the amplification program. To avoid false-positive results, usual precautions for PCR were strictly followed in the laboratory (*20*,*21*).

The threshold of detection of the nRT-PCR method was determined by preparing 10-fold dilutions of a pretitrated suspension of Strain 8918FRA (*4*) in TRIzol (GIBCO-BRL). Total RNA extraction, cDNA synthesis, and the RT-PCR procedures were performed as described above.

Sequencing of amplified products was performed by using the primers N62 and N63 and an Applied Biosystems 373A sequencer (Foster City, CA), according to the Applied Biosystems protocol. Multiple sequence alignments were generated with the Clustal W 1.60 program (*22*).

## Results

### Presence of EBL1 Antibodies in Six Bat Colonies

We describe here a very efficient technique of blood collection, which is more humane than collection by cardiac puncture (*23*,*24*). The bats recaptured 1 week after the blood extraction did not show any trace of a scar. Furthermore, our technique is easier than collection by puncture of the uropatagium or the propatagium cardiac veins (*25*). To eliminate any false- or doubtful positive reactions in seroneutralization, the threshold of positivity (titer=27) was chosen higher than the one adopted by other authors (*24*). Two independent repetitions of the seroneutralization also reinforced the accuracy of our results.

Throughout the 9-year study, 976 sera obtained from 14 bat species in 37 different locations were analyzed (Table 1); 76 (7.8%) were positive (Table 2). *Lyssavirus* antibodies were detected in four bat species (*Myotis myotis,* *Miniopterus schreibersii, Tadarida teniotis,* and *Rhinolophus ferrumequinum*). Sixteen positive sera and 5 negative sera against EBL1 (genotype 5 of lyssaviruses) were further tested against standard strains of genotypes 1 (CVS), and 6 (EBL2). These sera were obtained from the four EBL1-seropositive bat species and from another bat species that remained negative (*R. euryale*). None of them reacted positively against CVS and EBL2, confirming the specificity of the positive reactions against EBL1 obtained in these species (Table 3).

**Table 1:** Number of bat samples analyzed per species, 1992–2000^a^

Species	1992	1993	1994	1995	1996	1997	1998	1999	2000	Total
										
*R. ferrumequinum*		8				9/3		11/1	30/3	58/7
										
*R. euryale*		6			10					16
										
*R. hipposideros*				16		0/1				16/1
										
*P. pipistrellus*	61	64	75	18	13/5	0/16	0/14	3/15		234/50
										
*P. kulhii*				1						1
										
*E. serotinus*	21		44	33/1	1					99/1
										
*M. myotis*		1		63	65/2	44	29/2	58/8	35/3	295/15
										
*M. blythi*		20	1	2						23
										
*M. nattereri*		1					0/1		0/1	1/2
										
*M. capaccinii*						3				3
										
*M. emarginatus*				9		7/2				16/2
										
*P. austriacus*			3	6	2/4			1		12/4
										
*M. schreibersii*	8	18			14	8	9/2	70/6	41/1	168/9
										
*T. teniotis*					22	12				34
										
										
Total	90	118	123	148/1	127/11	83/22	38/19	143/30	106/8	976/91

^a^Where fractions (x/y) are shown, the numerator (x) corresponds to the number of sera analyzed and the denominator (y) to the number of brains analyzed. E = *Eptesicus*; M = *Myotis*; P = *Plecotus*; R = *Rhinolophus*; T = *Tadarida*.

**Table 2:** Positive serologic results in bat populations, the Spanish Autonomous Regions of Balearic Islands and Aragon, 1995–2000

Location and coordinates	Variables analyzed	1995	1996	1997		1998		1999	2000
No. 1	A/B^a^	-	-	1/5		-		0/11	1/20
	X±SD^b^	-	-	515		-		-	34
39°58’N,3°58’E	Species^c^	Rf	Rf	Rf		Rf		Rf	Rf
No. 3	A/B^a^							1/34	0/31
	X±SD^b^							215	
39°58’N,3°59’E	Species^c^	Ms	Ms	Ms		Ms		Ms	Ms
No. 4	A/B^a^	1/30	16/27	11/27		7/22		3/30	3/29
	X±SD^b^	90	348±237	191±225		718±657		78±27	58±42
39°44’N,2°58’E	Range		49–908	29-783		79-1677		47-95	29–107
	Species^c^	Mm	Mm	Mm		Mm		Mm	Mm
							
No. 5	A/B^a^	7/21	7/32	3/17	0/6	3/7	1/8	5/28	0/6
	X±SD^b^	122±45	207±159	218±136		412±454	8,508	106±61	
39°25’N,2°55’E	Range	83-195	53-442	129-374		87-930		29-176	
	Species^c^	Mm	Mm	Mm	Ms	Mm	Ms	Mm	Mm
							
No. 6	A/B^a^		0/22	2/12					
	X±SD^b^			243±284					
41°01’N,0°39’W	Range			420-444					
	Species^c^	Tt	Tt	Tt		Tt		Tt	Tt
							
No. 7	A/B^a^		2/14					2/19	
	X±SD^b^		93±68					35±6	
39°50’N,3°00’E	Range		45-141					31-40	
	Species^c^	Ms	Ms	Ms		Ms		Ms	Ms

^a^A = no. bats positive, B = no. bats analyzed. ^b^X = seroneutralization average; SD = standard deviation. *^c^Species analyzed: Rf* *=* Rhinolophus ferrumequinum*; Ms* *=* Miniopterus schreibersii*; Mm* *=* Myotis myotis*; Tt =* Tadarida teniotis*.*

**Table 3:** Specificity of results from serologic studies of bat populations, Spanish Autonomous Regions of Balearic Islands and Aragon, 1995–2000^a^

Location	Species	CVS	EBL1	EBL2
No. 1	*Rhinolophus ferrumequinum*	0 0	51 <27	ND ND
No. 4	*Myotis myotis*	0 0 0 0 0 0 0 0	588 222 350 246 709 <27 537 95	<27 <27 <27 <27 <27 ND <27 <27
No. 5	*M. myotis*	0 ND 0 0	53 421 97 188	<27 <27 <27 <27
				
No. 6	*Tadarida teniotis*	0 0 0	42 444 <27	ND ND ND
				
No. 7	*Miniopterus schreibersii*	0 0 0	45 141 <27	<27 <27 <27
				
	*Rhinolophus euryale*	0	<27	<27

^a^ND = not done; CVS = challenge virus strain**;** EBL1 = *European bat lyssavirus 1*.

The highest percentages of seropositive bats, 22.7% and 20.8%, were observed in the Balearic Islands in the locations of Inca (No. 4) and Llucmajor (No. 5), respectively (Table 2). From spring to autumn, location No. 4 shelters a plurispecific colony of approximately 1,000 bats belonging to the following species: *M. myotis* (25% of seropositives), *M. schreibersii*, *R. ferrumequinum*, *M. capaccinii,* and *M. nattereri*. At the beginning of summer, *M. myotis, M. nattereri,* and *M. schreibersii* species form breeding pairs. Location No. 5 shelters a summer-breeding colony of approximately 500 bats of the species *M. myotis* (22.5% of seropositives), *M. schreibersii* (7.1% of seropositives), and *M. capaccinii*. In both sites the most abundant species is *M. myotis*.

Seropositive bats were also found in four other locations, Nos. 1, 3, 6, and 7. Location No. 1 (5.5% of seropositive *R. ferrumequinum*) shelters a breeding colony of *R. ferrumequinum*. In spring, the colony also includes some *M. schreibersii*. Location No. 3 (2.9% of seropositive *M. schreibersii*) is a hibernation refuge for approximately 2,200 *M. schreibersii;* some *M. capaccinii* are also present. Location No. 6 (5.8% of seropositive *T. teniotis*) is a big sinkhole with a resident bat colony belonging to the following species: *T. teniotis*, *M. blythii*, *M. daubentonii*, *Pipistrellus pipistrellus*, *Pipistrellus kuhlii*, *Hypsugo savii*, *E. serotinus*, *Plecotus austriacus,* and *Barbastella barbastellus* (*26*). Location No. 7 (12% of seropositive *M. schreibersii*) shelters a colony of *M. schreibersii*, *M. capaccinii,* and *M. myotis*.

### Evolution of the Percentage of Seropositive Bats in Colonies Nos. 4 and 5

In Location 4, the percentage of seropositive bats rose from 3.3% in 1995 to 59.3% in 1996 (Table 2). Then it decreased significantly (Y=-15.6X + 31,196.5, r=-0.989, p<0.05) until 1999, when it reached 10%. This percentage remained stable in 2000. The percentage of seropositive bats remained stable in Location No. 5 from 1995 to 2000.

### Exchange of Animals Between Colonies and Survival of Seropositive Bats

During the period 1996-2000, 355 and 87 *M. myotis* were banded in Locations Nos. 4 and 5, respectively (Table 4). Recapture of the banded *M. myotis* allowed us to prove a few exchange of bats between the colonies. Two percent of *M. myotis* banded in Location No. 5 moved to Location No. 4 (the refuges are about 35 km apart). During the same period, 13 and 33 *M. schreibersii* were banded in Locations Nos. 5 and 7, respectively. One of the 33 *M. schreibersii* moved to Location No. 5 (the refuges are approximately 47 km apart); another moved to Location No. 4 (a distance of 11 km) (Figure 1).

**Table 4:** No. of recaptured and analyzed bats in Locations 4, 5, and 7, Spain, 1996–2000

Species^a^	BB^b^	BA^c^	BR^d^	BRD^e^	ATT^f^
Mm	442	221	25	2	4
Ms	46	46	0	2	1

^a^Species studied: Mm = *Myotis myotis*; Ms = *Miniopterus schreibersii*. ^b^BB = No.of bats banded. ^c^BA = No. of bats banded and analyzed. ^d^BR = No. of bats banded and recaptured in the same location. ^e^BRD = No. of bats banded and recaptured in different localities. ^f^ATT = No. of bats analyzed twice at interval of ≥ 1 year.

Banding also allowed us to follow the seroneutralization titer of some bats during the study period. The serum of a *M. schreibersii* captured in Location No. 7 in 1996 was negative; another serologic sample obtained from the same bat 2 years later in Location No. 5 yielded a titer of 8,508. During spring 2000, 12 *M. myotis* previously banded and analyzed were recaptured in Location No. 4. Four (33%) of them had already been shown to be seropositive in preceding years: two in summer 1997 (titers 29 and 145, respectively), one in summer 1998 (titer 303), and one in summer 1999 (titer 95). This indicates that some seropositive bats may survive at least 3 years after *Lyssavirus* infection.

### Detection and Characterization of EBL1 RNA in Bats

**Figure 2:**
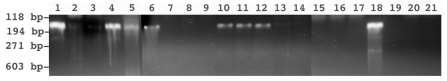
Detection of *European bat Lyssavirus 1* RNA in bats by nested reverse transcription-polymerase chain reaction (PCR). Lanes: 1, brain of *Miniopterus schreibersii* No. 140; 2- 5, medulla, tongue, esophagus-larynx-pharynx, and lung of *Rhinolophus ferrumequinum* No. 135, respectively; 6-14, brain, medulla, esophagus-larynx-pharynx, liver, lung, heart, tongue, spleen, and kidney of *R. ferrumequinum* No. 128, respectively; 15, negative control of RNA extraction of bat No. 135; 16, negative control of RNA extraction of bat No. 128; 17, negative control of RNA extraction of bat No. 140; 18, positive control; 19, negative control of first PCR; 20, negative control of second PCR.

During 1995 through 1996, 12 brain samples were only analyzed by FAT. After 1996, the brain samples (n=79) were also analyzed by nested RT-PCR (Table 1). All brains (n=91) analyzed by FAT were negative. In contrast, brains of 1 *M. myotis*, 1 *M. nattereri,* and 1 *M. schreibersii* (No. 140) of Location No. 4 and 1 *R. ferrumequinum* (No. 128) of Location No. 1 (all collected in 2000) were positive by nested RT-PCR. Four animals (*M. schreibersii* [No. 140] and *R. ferrumequinum* [No. 128], whose brains were positive by nested RT-PCR, and two *R. ferrumequinum* [No. 123 and No. 135], whose brains were negative) were completely necropsied. Various organs and tissues (medulla, liver, kidney, spleen, heart, tongue, esophagus-larynx-pharynx, and lung) were collected and subjected to nRT-PCR. Esophagus-larynx-pharynx and lung of bat No. 135 and tongue, lung, and heart of bat No. 128 were positive (Figure 2).

Twenty-seven blood pellets of bats collected in 2000 were also analyzed by nRT-PCR. These samples were obtained from 8 *R. ferrumequinum* (location No. 1), 1 *R. ferrumequinum* (Location No. 3), 1 *M. myotis* (Location No. 5), 14 *M. myotis* (Location No. 4), and 3 *M. schreibersii* (Location No. 4). The blood pellets of three *M. myotis* from Location No. 4 were found positive by nRT-PCR. None of the blood samples showing positive RT-PCR results on the pellet were found positive by seroneutralization.

The threshold of detection of the nRT-PCR for the amplification of the EBL1a genomic and antigenomic RNAs of the N gene was 5 x 10^-2^ fluorescent forming units of EBL1a/mL. In all these experiments, negative controls performed individually for each step (extraction, RT, primary, and secondary PCR) were negative. Furthermore, nRT-PCR performed on positive tissues without previous reverse transcription gave negative results, demonstrating the absence of complementary DNA contamination.

Nucleotide (nt) sequences were determined by using the positive nRT-PCR products obtained from the four brains and from one blood sample. These 122-nt long sequences of the nucleoprotein gene were strictly similar to the sequence of two EBL1b Spanish isolates (94285SPA and 9483 SPA) described previously (*5*), except that the sequence obtained from the positive blood pellet exhibited a T→A mutation in position 145 of the coding region of the nucleoprotein gene. Four mutations distinguished the sequence of the positive control corresponding to a French bat (No. 2002FRA) from the different sequences obtained from Spanish bats (not shown). This further confirms the specificity of the products amplified from the Spanish bat samples.

## Discussion

This is the first report of the presence of EBL1-specific neutralizing antibodies in four European insectivorous bat species (*M. myotis,* *M. schreibersii, T. teniotis,* and *R. ferrumequinum*). These findings lead to the following observations on the circulation and possible bat species involved in the dispersion of EBL1 in southern Europe. First, the identification of EBL1 antibodies in 24% of the *M. myotis* analyzed in Locations No. 4 and No. 5 in 1995 through 2000 (n=276) indicates that bats of this genus are infected with EBL1. Second, the distribution of *T. teniotis* and *M. schreibersii* in southern Europe and northern Africa (*13*,*27*) could contribute to the dispersion of EBL1 in southern Europe and is concordant with the possible African origin of EBL1, as suggested by Amengual et al. (*5*).

Although the seasonal movements of *T. teniotis* are scarcely known, the quick, straight flight of this species suggests that such movements are long, as is the case with the American bat (*T. brasiliensis mexicana),* which is capable of performing annual migrations of more than 1,000 km. Since *M. schreibersii* makes seasonal migrations (some of them >350 km) (*16*), this species could also be one of the dispersion vectors of the disease in southern Europe, where it abounds. *M. schreibersii* dwells in five out of the six sites where seropositive bats have been found. In three of them, *M. schreibersii* forms mixed colonies with *M. myotis*, in one it shelters next to *R. ferrumequinum,* and in the fifth it shelters alone. *M. schreibersii* and *M. myotis* have direct physical contact in the mixed colonies. However, it is unlikely that *Pipistrellus nathusii* is a dispersion vector of the lyssaviruses in Spain, as Brosset (*6*) suggests, since this is a very rare bat in the Iberian Peninsula.

**Figure 3:**
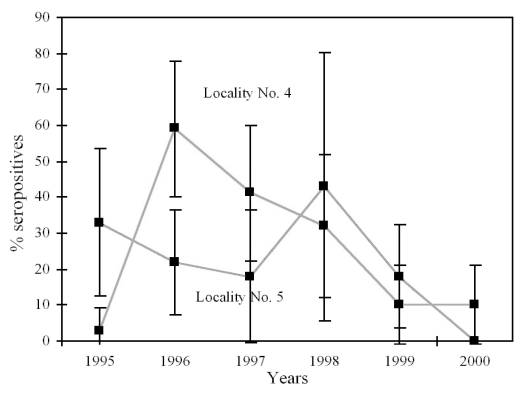
Incidence of seropositive bats observed in *Myotis myotis* colonies, Spanish Locations No. 4 and No. 5, 1995–2000 (95% confidence intervals shown).

The results obtained in 1995-2000 in Location No. 4 show that the evolution in the number of seropositive bats after a *Lyssavirus* infection corresponded to an asymmetrical curve, with a sudden initial increase reaching more than 60% of the colony and a gradual decline over subsequent years (*24*)—unless a new episode took place (Figure 3). Because of the gregarious behavior of this species, a quick increase and a high seropositive percentage (almost 60% in this location) after a *Lyssavirus* episode are not unusual. The intimate contact that always exists among bats must facilitate viral transmission and antibody development. A high seropositive percentage also occurs in colonies of *T. brasiliensis mexicana*, where percentages >80% have been observed (*10*,*11*). The transmission of lyssaviruses between bats from mixed colonies could take place through breathing or biting but is currently not documented.

The low prevalence (0 of 91, <1.1%) of active infection as determined by FAT is concordant with previous results obtained in America, which show a prevalence of active rabies infection in bats between 0.1 and 2.9% (*10**,**28**,**29*). However, we report the first detection of EBL1 RNA by nRT-PCR in several tissues (brain, blood pellet, lung, heart, tongue, and esophagus-larynx-pharynx) of four *M. myotis*, one *M. nattereri*, one *M. schreibersii,* and two *R. ferrumequinum*. These isolates show the existence of a low or nonproductive infection in these species, although some small remnant of RNA remaining in a clinically normal bat as a result of an earlier nonlethal exposure to a *Lyssavirus* is also possible*.* This low amount of viral DNA present in the tissues underscores the need to use nRT-PCR as a very sensitive technique for epidemiologic studies of EBL1 in bat populations. Rønsholt et al. (*8*) also comment on the difficulty of detecting *Lyssavirus* infection by immunofluorescence in bats when a clinically silent infection exists.

EBL1 are known to actively infect the brain, lung, and tongue of *E. serotinus* (*3*). However, this is the first report that EBL1 RNA can be detected in various organs and tissues in the absence of active infection, as demonstrated by negative results obtained by FAT. Most of these bats were dead when collected but were kept in conditions that allowed the classic diagnosis by FAT to be performed properly. These negative FAT results indicate that these bats died of causes other than their low productive *Lyssaviru*s infection. The recapture of seropositive bats over several years also shows that some of these bats survived EBL1 infection. The detection of EBL1b sequences in the blood pellet of bats (3/27) is also a new finding. This technique would be an easy test for screening positive bats. However, further studies are needed to establish the interest and sensitivity of this sample.

The sensitivity of the different European bat species to EBL infection probably varies according to the animal and virus species involved. Therefore, we have summarized in Table 5 (*2*,*5*,*24*,*30*,*31*) the bat species in which either *Lyssavirus* or antibodies against *Lyssavirus* have been detected. Further studies are needed to determine which of the European bat species are the reservoir of EBL infection and if different species act as sentinels for the presence of the virus in the colony.

**Table 5:** Bat species positive for *Lyssavirus,* Europe, 1954–2000^a^

Family	*Species*	*Lyssavirus ^b^*	Antibodies *^c^*
*Vespertilionidae*	*Eptesicus serotinus*	EBL1a & b	EBL1
	*Pipistrellus pipistrellus*	NC	ND
	*Pipistrellus nathusii*	NC	ND
	*Vespertilio murinus*	EBL1a	ND
	*Myotis dasycneme*	EBL2a	ND
	*Myotis daubentonii*	EBL2a & b	ND
	*Myotis myotis*	EBL1b	EBL1
	*Myotis nattereri*	EBL1b	ND
	*Nyctalus noctula*	NC	ND
	*Miniopterus schreibersii*	EBL1b	EBL1
*Molossidae*	*Tadarida teniotis*	NC	EBL1
*Rhinolophidae*	*Rhinolophus ferrumequinum*	EBL1b	EBL1

^a^The additional information was obtained from Kappeler (*29*), Pérez-Jordá et al. (*24*), Amengual et al. (*5*), Bulletin épidemiologique mensuel de la rage en France (*30*), and Muller (*2*). ^b^NC = not characterized. ^c^ND = not done.

The presence of EBL1 RNA and immunity to EBL1 in several wild bat colonies also has important implications for bat management and public health. The probability of humans’ having contact with these colonies should be reduced and controlled. In our study, most bat colonies were found in sites that are frequently visited by speleologists, tourists, and bat-lovers. As a consequence of our findings, the entry to these caves is now controlled and limited during the periods when bats are present (in spring, summer, and autumn for Location No. 4). Entry is limited by horizontal bars that allow the bats to fly across them but prevent access to people without obscuring the view.
